# MicroRNAs in cell proliferation, cell death, and tumorigenesis

**DOI:** 10.1038/sj.bjc.6603023

**Published:** 2006-02-21

**Authors:** H-W Hwang, J T Mendell

**Affiliations:** 1 Program in Human Genetics and Molecular Biology, The Institute of Genetic Medicine, Johns Hopkins University School of Medicine, 733 N Broadway, BRB 460C, Baltimore, MD 21205, USA

**Keywords:** microRNAs, cellular proliferation, apoptosis, oncogene, tumour suppressor

## Abstract

MicroRNAs (miRNAs) are a recently discovered class of ∼18–24 nucleotide RNA molecules that negatively regulate target mRNAs. All studied multicellular eukaryotes utilise miRNAs to regulate basic cellular functions including proliferation, differentiation, and death. It is now apparent that abnormal miRNA expression is a common feature of human malignancies. In this review, we will discuss how miRNAs influence tumorigenesis by acting as oncogenes and tumour suppressors.

MicroRNAs (miRNAs) were first discovered in the early 1990s by Ambros, Ruvkun, and co-workers while studying development in the nematode *Caenorhabditis elegans* (Lee *et al*, 1993; Wightman *et al*, 1993). Their analyses of genes that control developmental timing revealed that *lin-14* was necessary for the differentiation of specific cell lineages and *lin-4* might be a *trans*-acting negative regulator of *lin-14*. Subsequently, it was determined that *lin-4* encodes a 22-nucleotide noncoding RNA which negatively regulates the translation of *lin-14* by directly base pairing to complementary sites within its 3′-untranslated region (3′ UTR). Since very few examples of regulation of an mRNA by a naturally occurring antisense RNA had been described in eukaryotes, this form of regulation was generally considered a fascinating, but likely worm-specific, oddity. Seven years later, another small regulatory RNA, *let-7*, was identified as an additional regulator of developmental timing in *C. elegans* (Reinhart *et al*, 2000). Strikingly, both the sequence and temporal expression pattern of *let-7* were conserved in a wide variety of animals (Pasquinelli *et al*, 2000), suggesting a more general role for small RNAs in gene regulation. Nevertheless, the broad significance of these findings was not fully appreciated until hundreds of similar small RNAs were identified in *C. elegans*, *Drosophila*, and mammals by molecular cloning and bioinformatics analyses in 2001 (reviewed in Bartel, 2004). These newly discovered ∼18–24 nucleotide RNAs were termed microRNAs (miRNAs). Since their initial description, more than 3000 miRNAs have been identified in animals, plants, and viruses. In the human genome, more than 300 miRNAs have been discovered, and the estimated number of miRNA genes is as high as 1000 (Bentwich *et al*, 2005; Berezikov *et al*, 2005). As a group, miRNAs are estimated to regulate 30% of genes in the human genome (Lewis *et al*, 2005).

In addition to providing critical functions during normal development and cellular homeostasis, it has become increasingly clear that abnormalities in miRNA activity contribute to human disease pathogenesis. In particular, an important role for miRNAs in the development of cancer has emerged. In this review, we will discuss the functions of cancer-relevant miRNAs that regulate cellular proliferation, death, and tumorigenesis.

## MIRNA BIOGENESIS AND FUNCTION

MicroRNAs are initially transcribed by RNA polymerase II (pol II) as long primary transcripts known as primary-miRNAs (pri-miRNAs). Like other pol II transcripts, these RNAs are capped, polyadenylated, and are usually several kilobases in length (reviewed in Kim, 2005). Approximately half of pri-miRNAs appear to lack significant open reading frames and are likely noncoding RNAs. Nevertheless, these transcripts are often spliced and many miRNAs are located within their intronic segments. Alternatively, a significant fraction of miRNAs (∼40%) are found within introns of known protein-coding genes (Rodriguez *et al*, 2004). Additionally, miRNAs are frequently clustered such that a single pri-miRNA contains multiple miRNA sequences.

**Figure 1:**
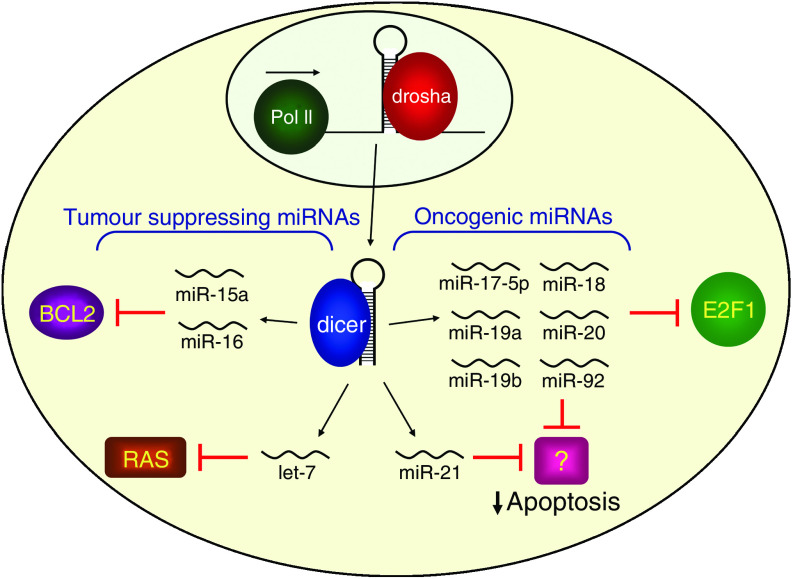
The biogenesis and functions of cancer-associated human miRNAs. A simplified schematic of miRNA biogenesis is shown. MicroRNAs are initially transcribed by RNA polymerase II (Pol II) and sequentially processed by drosha and dicer (see text for details). MicroRNAs with tumour suppressing or oncogenic properties are shown with their experimentally validated targets.

Within the pri-miRNA, the miRNA itself is contained within a ∼60–80 nucleotide sequence that can fold back on itself to form a stem-loop hairpin structure. MicroRNA hairpins are recognised and excised from pri-miRNAs in the nucleus (Figure 1) by the microprocessor complex, which includes the RNase III enzyme drosha and its binding partner DGCR8 (Kim, 2005). The excised miRNA hairpins, referred to as pre-miRNAs, contain a two nucleotide 3′ overhang characteristic of RNase III cleavage products. Pre-miRNAs are rapidly exported to the cytoplasm by the nuclear export factor exportin 5 which uses Ran-GTP as a cofactor (Yi *et al*, 2003; Bohnsack *et al*, 2004; Lund *et al*, 2004). Further cytoplasmic processing by dicer, a second RNase III enzyme, produces an ∼18–24 nucleotide duplex. This fully processed duplex is incorporated in an ATP-independent manner into a large protein complex known as the RNA-induced silencing complex, or RISC, which includes as core components the Argonaute proteins (Ago1–4 in humans). Only one strand of the miRNA duplex remains stably associated with RISC. This strand becomes the mature miRNA. The opposite strand, known as the passenger strand or miRNA^*^, is disposed of through two alternative mechanisms. When miRNAs are loaded into RISC containing Ago2, the only human Ago protein capable of cleaving target mRNAs, the passenger strand may be cleaved. Alternatively, RISC containing any Ago protein may remove the passenger strand via a bypass mechanism that does not require cleavage and likely involves duplex unwinding (Gregory *et al*, 2005; Matranga *et al*, 2005; Rand *et al*, 2005).

MicroRNAs act as guides that direct RISC to target mRNAs that are subsequently cleaved or translationally silenced (reviewed in Bartel, 2004). The degree of complementarity between an miRNA and its target determines the mechanism of regulation. When an miRNA and an mRNA exhibit perfect complementarity, the target mRNA is cleaved by RISC. This appears to be the predominant mechanism through which miRNAs function in plants. Imperfect base pairing between an miRNA and its target, as occurs with most *C. elegans*, *Drosophila*, and mammalian miRNAs, leads to translational silencing of the target. However, imperfectly complementary miRNAs can also reduce the abundance of target mRNAs to varying degrees (He and Hannon, 2004).

## EARLY CLUES THAT MIRNAS REGULATE CANCER-RELEVANT PATHWAYS

Malignant cell growth is typified by loss of cellular identity, enhanced proliferation, and dysregulated cell death. Early clues that miRNAs were important in regulating these cancer-relevant cellular phenotypes came from genetic screens in *C. elegans* and *Drosophila*. As mentioned above, the first miRNA identified, *lin-4*, controls the timing of developmental events in *C. elegans.* Loss-of-function of *lin-4* results in the abnormal differentiation of specific cell lineages and the reiteration of embryonic cell types in later stages of development (Lee *et al*, 1993). These abnormalities are reminiscent of tumours which are caused by a failure to fully execute a programme of differentiation. For example, in many haematologic malignancies, a block in differentiation causes immature cells to accumulate with pathologic consequences.

In *Drosophila*, overexpression of the *Bantam* miRNA causes overgrowth of wing and eye tissue. It was subsequently demonstrated that *Bantam* stimulates cell proliferation and prevents apoptosis at least in part through negative regulation of the proapoptotic gene *hid* (Brennecke *et al*, 2003). Another *Drosophila* miRNA, miR-14, was identified through its ability to suppress cell death. Flies harbouring a deletion of this miRNA were found to have an increased expression of an apoptotic effector caspase, *Drice*, which has a putative miR-14 target site (Xu *et al*, 2003). However, direct regulation of *Drice* by miR-14 remains to be demonstrated. In another study, antisense-mediated inhibition of the miR-2/6/11/13/308 family in *Drosophila* embryos was demonstrated to induce widespread apoptosis. These miRNAs were subsequently shown to regulate the proapoptotic factors *hid, grim, reaper*, and *sickle* (Leaman *et al*, 2005).

## MIRNA DYSREGULATION IN CANCER

A more direct link between miRNA function and cancer pathogenesis is supported by studies examining the expression of miRNAs in clinical samples. The first study documenting abnormalities in miRNA expression in tumour samples focused on B-cell chronic lymphocytic leukaemia (B-CLL). Deletion of chromosome 13q14 is the most frequent chromosomal abnormality in this disorder and it has been postulated that a tumour suppressor gene resides in this region. Croce and co-workers demonstrated that this tumour suppressor activity is likely provided by two miRNAs, miR-15a and miR-16-1, which are localised in the minimally deleted region and are frequently deleted or downregulated in CLL patients (Calin *et al*, 2002). More recently, these investigators found that a specific expression signature consisting of 13 miRNAs, including miR-15a and miR-16-1, was associated with disease progression in CLL (Calin *et al*, 2005).

More than half of miRNAs are located at sites in the human genome that are frequently amplified, deleted, or rearranged in cancer, suggesting that miRNA abnormalities play a broad role in cancer pathogenesis (Calin *et al*, 2004). Also consistent with this notion is the observed dysregulation of miRNA expression in diverse cancer subtypes including Burkitt's lymphoma (Metzler *et al*, 2004), colorectal cancer (Michael *et al*, 2003), lung cancer (Takamizawa *et al*, 2004), breast cancer (Iorio *et al*, 2005), and glioblastoma (Chan *et al*, 2005).

Recently, Golub and co-workers used a bead-based flow cytometric method to profile 217 mammalian miRNAs across a large panel of samples representing diverse human tissues and tumours (Lu *et al*, 2005). They found that miRNA profiles were highly informative, reflecting the developmental lineage and differentiation state of the tumours. MicroRNA profiles were used to identify the tissue origin of poorly differentiated tumours with greater accuracy than profiles constructed using ∼16 000 mRNAs. Furthermore, tumours within the same lineage harbouring distinct chromosomal rearrangements were found to exhibit distinct miRNA expression profiles. Thus, the developmental history of a tumour is reflected in its miRNA expression pattern.

Irrespective of cell type, more than half of the miRNAs examined in the Golub study were expressed at significantly lower levels in tumours compared with normal tissues. This likely reflects a role for miRNAs in terminal differentiation and the relatively incomplete differentiation status of cancer cells. Consistent with this hypothesis, inducing differentiation of a myeloid leukaemia cell line with all-*trans* retinoic acid increased the expression of many miRNAs. A similar induction of miRNA expression was also observed in primary human haematopoietic progenitor cells undergoing erythroid differentiation.

The emerging view from these studies is that dysregulation of miRNA expression is a frequent occurrence in diverse types of cancer. These findings highlight the potential utility of miRNA profiling for diagnostic and prognostic applications. These studies also highlight a need for more direct functional analyses of the roles of miRNAs in regulating pathways relevant to tumour pathogenesis. These types of experiments are now beginning to be carried out, as described below.

## PROPROLIFERATIVE/ANTIAPOPTOTIC MIRNAS

MicroRNAs with proproliferative and antiapoptotic activity would likely promote oncogenesis and thus may be overexpressed in cancer cells. The most comprehensively studied example of an miRNA locus with these properties is the *mir-17* cluster, consisting of six miRNAs: miRs-17-5p, -18, -19a, -19b, -20, and -92. This miRNA cluster is located on human chromosome 13q31, a region that is frequently amplified in several types of lymphoma and solid tumours (Ota *et al*, 2004). In a recent study, He *et al* (2005) found that miRNAs from this cluster are highly expressed in B-cell lymphoma samples and cell lines. To directly interrogate the tumorigenic properties of these miRNAs, they utilised E*μ*-Myc transgenic mice, a model of B-cell lymphoma in which expression of the proto-oncogene c-Myc is driven by the immunoglobulin heavy-chain enhancer. c-Myc encodes a helix-loop-helix transcription factor that is abnormally active in a large fraction of human malignancies. Elevated c-Myc expression accelerates cell proliferation and growth and, in some settings, induces apoptosis. E*μ*-Myc transgenic mice develop B-cell lymphomas relatively late in life (4–6 months of age) and with incomplete penetrance. When a truncated form of the *mir-17* cluster (lacking miR-92) was introduced into haematopoietic stem cells from these mice, disease onset was dramatically accelerated. Whereas E*μ*-Myc lymphomas are highly apopotic, the miRNA-expressing lymphomas show a high mitotic index without extensive apoptosis, suggesting that these miRNAs act primarily by suppressing cell death. Interestingly, when each of the miRNAs of the *mir-17* cluster was individually coexpressed with c-Myc, none accelerated tumour formation, suggesting that the oncogenic effect requires a cooperative interaction between the miRNAs in the cluster. Moreover, no increased lymphomagenesis was observed after expressing 96 different single miRNAs in this model, demonstrating the specificity of the *mir-17* cluster finding.

While a functional interaction between the *mir-17* cluster and Myc was being explored in the E*μ*-Myc animals, independent work by O'Donnell *et al* (2005) discovered that these miRNAs were directly regulated at the transcriptional level by c-Myc. In multiple cellular models, induction of c-Myc expression correlated with induction of the *mir-17* cluster. Furthermore, c-Myc was found to bind directly to the genomic locus encoding these miRNAs to activate their transcription. The authors went on to show that two miRNAs in this cluster, miR-17-5p and miR-20, regulate the translational efficiency of the proproliferative/proapoptotic transcription factor E2F1. Interestingly, E2F1 is known to be transcriptionally activated by c-Myc (Fernandez *et al*, 2003). Since high expression of E2F1 is sufficient to induce apoptosis in some settings, simultaneous transcriptional activation by c-Myc and dampening of translational efficiency by the *mir-17* cluster may tune E2F1 activity such that cell death is disfavoured and proliferation is promoted. This hypothesis is consistent with the decreased apoptosis observed in the E*μ*-Myc miRNA-expressing tumours.

Amplification of the *mir-17* cluster and overexpression of these miRNAs has more recently been observed in lung cancers and in particular small-cell lung cancer (Hayashita *et al*, 2005). In a lung cancer cell line, overexpression of the entire *mir-17* cluster, but not individual miRNAs from the cluster, accelerated cell proliferation. These findings further document the oncogenic properties of this group of miRNAs.

Another miRNA with an apparent antiapoptotic function is miR-21. This miRNA was found to be overexpressed in glioblastoma tumour tissues and cell lines (Chan *et al*, 2005). High expression of this miRNA was also observed in breast cancer tissue (Iorio *et al*, 2005). Inhibition of miR-21 in glioblastoma cell lines using 2′-*O*-methyl antisense oligonucleotides, which are known to inhibit miRNA function, resulted in caspase activation and increased apoptosis (Chan *et al*, 2005).

## ANTIPROLIFERATIVE/PROAPOPTOTIC MIRNAS

MicroRNAs with antiproliferative and proapoptotic activity are likely to function as tumour suppressor genes and thus may be underexpressed in cancer cells. The family of *let-7* miRNAs represents a clear example of this. The *let-7* family consists of a group of highly conserved miRNAs in multiple species including *C. elegans*, *Drosophila*, and vertebrates. *let-60/RAS* was recently identified as a target of *let-7* in *C. elegans* (Johnson *et al*, 2005). This regulation appears to be conserved in humans where three RAS genes, known to be potent oncogenes, have also been demonstrated to be directly regulated by human let-7. Since RAS dysregulation is a key oncogenic event in lung cancer, loss of let-7 may contribute to pathogenesis in this disorder. Indeed, let-7 is generally expressed at low levels in cancerous lung tissue compared to normal tissue and low expression of this miRNA correlates with shorter postoperative lung cancer survival (Takamizawa *et al*, 2004). Furthermore, in samples from three lung cancer patients, RAS protein levels, but not RAS mRNA levels, were inversely correlated with let-7 levels, consistent with miRNA-mediated translational repression (Johnson *et al*, 2005). Expression of let-7 in lung cancer cell lines directly suppresses growth *in vitro*, further documenting the tumour suppressing activity of this miRNA (Takamizawa *et al*, 2004).

miR-15a and miR-16-1 represent additional miRNAs with likely tumour suppressing activity. As mentioned above, these miRNAs are located on human chromosome 13 in a region frequently deleted in B-CLL. Cimmino *et al* (2005) identified a conserved target site for miR-15a and miR-16-1 in the 3′ UTR of BCL2, a potent inhibitor of cell death. Consistent with a regulatory interaction between these miRNAs and this gene, the levels of miR-15a and miR-16-1 are inversely correlated with Bcl2 protein levels in samples from CLL patients. Furthermore, overexpression of miR-15a and miR-16-1 in a leukaemic cell line results in decreased Bcl2 protein expression, activation of the intrinsic apoptosis pathway, and ultimately cell death. Thus, loss of these miRNAs may contribute to elevated Bcl2 expression and pathologic cell survival in B-CLL.

## MIRNAS IN CELL CYCLE PROGRESSION AND STEM CELL MAINTENANCE

Both cancer cells and stem cells share an ability to divide continuously without undergoing senescence. Additionally, evidence supports the existence of ‘cancer stem cells’ that may be important in the genesis of certain types of cancers including leukaemia and solid tumours. Because of the importance of these cells in normal and pathophysiologic states, there is growing interest in elucidating the mechanisms that confer the unique properties of these cells. Recent experiments in which components essential for miRNA biogenesis were disrupted in flies suggest that miRNAs may play a role in stem cell maintenance. *Drosophila* with a null mutation of dicer-1 (dcr-1), which is required for miRNA processing, exhibited abnormalities in germline stem cell (GSC) function (Hatfield *et al*, 2005). Specifically, dcr-1-deficient GSCs inappropriately activated the G1/S cell cycle checkpoint, suggesting that miRNAs normally act to promote continuous cell cycle progression. This cell cycle delay was specific to GSCs and did not occur in other tissues such as imaginal discs. Reduction of expression of Dacapo (Dap), a *Drosophila* homologue of the p21/p27 family of cyclin-dependent kinase inhibitors, partially rescued the dcr-1 deficiency phenotype. Dap is predicted to be regulated by multiple miRNAs and expression of a Dap transgene lacking these putative miRNA-binding sites mimicked the phenotype of dcr-1 loss-of-function. Taken together, these data suggest that miRNAs normally function to suppress Dap expression in GSCs, thus allowing cell-cycle progression.

## CONCLUDING REMARKS

It is now well established that miRNAs play essential roles in many basic physiologic processes including proliferation, differentiation, and apoptosis. Abnormalities in miRNA function impinge upon the normal regulation of these pathways and thus influence tumorigenesis. Undoubtedly, the growing interest in this class of regulatory RNAs will lead to continued classification of miRNA expression in cancer samples and the identification of new miRNAs that may act as oncogenes and tumour suppressors. The key to understanding the roles of these molecules in cancer will be identifying and validating the critical targets responsible for the phenotypic effects of miRNA loss- or gain-of-function. Although bioinformatics approaches have produced exhaustive lists of putative targets, verifying these regulatory interactions and determining which are the critical targets in a given tissue or tumour remains arduous.

Another potential challenge for future studies relates to the probable tissue-specific functions of some miRNAs. The function of a given miRNA is dictated by the milieu of targets that are expressed in a given cell type. Thus, an miRNA that regulates both proproliferative and antiproliferative targets may act as a tumour suppressor in some cancers and an oncogene in others, depending on which targets are driving tumorigenesis. One example of this may be the *mir-17* cluster. The evidence that these miRNAs have oncogenic activity was presented earlier in this review. Nevertheless, the chromosomal locus encompassing these miRNAs is known to undergo loss-of-heterozygosity in hepatocellular carcinoma (Lin *et al*, 1999), suggesting that these miRNAs may have tumour suppressor activity in this type of cancer. Additionally, many proproliferative targets are predicted to be downregulated by these miRNAs. Future studies are necessary to determine if these and other miRNAs have cell-type-specific effects on cell proliferation and death.

Although we are at an early stage in our understanding of the roles of miRNAs in cancer, the importance of these molecules in this disease process are clear. Undoubtedly, continued efforts to delineate miRNA function in physiologic and pathophysiologic states will reveal novel insights into normal cellular and developmental biology and the mechanisms that are disrupted when these processes go awry.
